# Supplementary material for: EEG-Based Tool for Prediction of University Students’ Cognitive Performance in the Classroom
Source: Brain Sci. 2021 May 26;11(6):698. doi: 10.3390/brainsci11060698 (PMC8227309; doi:10.3390/brainsci11060698)
Supplement: Supplementary file 1 [file brainsci-11-00698-s001.zip › Supplementary Material/Evaluation English.pdf]

- **First test**

1. What is CAN Bus?
    a. Interference communication protocol
    b. Communication protocol between electronic modules
    c. Communication data transport protocol.

2. It is an advantage of using CAN bus
    a. Reduces the use of wires
    b. It has filters that increase noise
    c. It has no advantages

3. Select at least one type of information that travels through this network
    a. Telephone data
    b. Temperature data
    c. Environmental data

- **Second test**

1. What does CAN bus allow?
    a. Transport of a big amount of data through several wires
    b. Transport of a big amount of data through one wire
    c. Transport of single data through several wires

2. What kind of information travels through this network?
    a. Information on temperature, window lift motor and oil pressure
    b. Information on battery, service and breaks
    c. Information on power, failures and diesel

3. What are the main components of CAN bus?
    a. Controller, multiplexed wires, terminator
    b. Starter, braided wires, finalizer
    c. Controller, multipolar wires, router

4. The presence of a decoder is an advantage
    a. True
    b. False

5. CAN bus is a communication protocol between _____________ modules
    a. Electrical
    b. Electronic
    c. Electromagnetic

- **Third test**

1. CAN bus is a communication protocol between ____________ modules
   a. Electrical
   b. Electronic
   c. Electromagnetic

2. The main aim of CAN bus is to transport a big amount of data through several wires
   a. True
   b. False

3. Component that receives, encodes and sends information:
   a. Terminator
   b. Controller
   c. Multiplexed wires

4. Match the correct options

It is used to close and adjust the function of the system	Terminador
Helps to filter electrical noise	Multiplexed wires
Receives, encodes and sends information	Controller

5. What percentage of wires gets reduced by CAN bus?

   a. Around 45%
   b. Around 55%
   c. Around 35%

6. CAN bus reduces electrical noise.
   a. True
   b. False

7. CAN bus is this type of signal:
   a. Serial and analogic
   b. Analogic and digital
   c. Serial and digital

8. What type of cable is used to avoid electrical noise or electromagnetic waves interfere with the signal?
   a. Insulating wires
   b. Shielded wires
   c. Braided wires

9. Match the correct option about the speed of sent information:

CAN_L	500kbits/s
CAN_H	125kbits/s
