# Supplementary material for: EEG-Based Tool for Prediction of University Students’ Cognitive Performance in the Classroom
Source: Brain Sci. 2021 May 26;11(6):698. doi: 10.3390/brainsci11060698 (PMC8227309; doi:10.3390/brainsci11060698)
Supplement: Supplementary file 1 [file brainsci-11-00698-s001.zip › Supplementary Material/Evaluation Spanish.pdf]

- **Primer cuestionario**

1. ¿Qué es la Red CAN?
    a. Protocolo de interferencia en la comunicación
    b. Protocolo de comunicación entre módulos electrónicos
    c. Protocolo de transporte datos de la comunicación

2. Menciona alguna ventaja de utilizarla
    a. Usar menos cableado
    b. Posee filtros que aumentan el ruido
    c. No tiene ventajas

3. Selecciona al menos una información que viaja por esta red
    a. Datos telefónicos
    b. Datos de temperatura
    c. Datos ambientales

- **Segundo cuestionario**

1. ¿Qué es lo que permite la Red CAN?
    a. Transportar gran cantidad de datos por infinidad de cables
    b. Transportar gran cantidad de datos por un solo cable
    c. Transportar por infinidad de cables un solo dato

2. ¿Qué tipo de información viaja por esta red?
    a. Información sobre temperatura, levanta cristales, presión de aceite
    b. Información sobre batería, servicio, frenos
    c. Información sobre potencia, fallas, diesel

3. ¿Cuáles son los principales componentes?
    a. Controlador, cables multiplexados, terminador
    b. Comenzador, cables trenzados, finalizador
    c. Controlador, cables multipolares, router

4. La presencia de un decodificador es una ventaja
    a. CIERTO
    b. FALSO

5. Red CAN es un protocolo de comunicación entre módulos
    a. Eléctricos
    b. Electrónicos
    c. Electromagnéticos

- **Tercer cuestionario**

1. Red CAN es un protocolo de comunicación entre módulos
    a. Eléctricos
    b. Electrónicos
    c. Electromagnéticos

2. El principal objetivo es transportar una gran cantidad de datos por infinidad de cables.
    a. Cierto
    b. Falso

3. Componente que recibe, codifica y envía la información:
    a. Terminador
    b. Controlador
    c. Cables multiplexados

4. Une con la opción correcta

| | |
|---|---|
| Se utiliza para cerrar y adecuar el funcionamiento del sistema | Terminador |
| Ayudan a filtrar el ruido eléctrico | Cables multiplexados |
| Recibe, codifica y envía la información | Controlador |

5. ¿Qué porcentaje aproximadamente de cables y conexiones llega a reducir?

    a. Alrededor del 45%
    b. Alrededor del 55%
    c. Alrededor del 35%

6. Red CAN reduce los ruidos eléctricos.
    a. Cierto
    b. Falso

7. Es un tipo señal…
    a. Serial y analógica
    b. Analógica y digital
    c. Serial y digital

8. ¿Qué tipo de cable se utiliza para evitar que el ruido eléctrico u ondas electromagnéticas interfieran en la señal?
    a. Cables aisladores
    b. Cables blindados
    c. Cables trenzados

9. Une con la opción de velocidad de información correcta:

| | |
|---|---|
| CAN_L | 500kbits/s |
| CAN_H | 125kbits/s |
