# Supplementary material for: EEG-Based Tool for Prediction of University Students’ Cognitive Performance in the Classroom
Source: Brain Sci. 2021 May 26;11(6):698. doi: 10.3390/brainsci11060698 (PMC8227309; doi:10.3390/brainsci11060698)
Supplement: Supplementary file 1 [file brainsci-11-00698-s001.zip › Supplementary Material/LT Text English.pdf]

CAN BUS

- All control units communicate through this network (Controller Area Network)

What is a CAN BUS? (Controller Area Network)

It is a communication protocol between electronic modules. This communication allows transporting a large amount of data over a single cable.

Who and when created the Network?

It was first incorporated into a vehicle in 1987 by Intel and Philips, and it is now included in all vehicles.

What are the components of the CAN Network?

- Controller (Transmitter and Receiver):

- These components are the modules (or computers).
- This circuit receives, encodes and sends information.
- Operate in very low current values, usually around 0.5 A.

- **Multiplexed cables:**

- Cables in charge of relaying this information.
- Filter the electrical noise.

- **Terminator:**

- Resistive elements used to close and adjust the system's operation.

This type of communication allows a connection between the different "computers" that the vehicle has in a very efficient way. This whole set is called a multiplexed communication system.

The connections must follow certain rules or protocols, this protocol is called CAN (Controller Area Network). Some information that travels through this network are:

- Engine oil pressure
- Lift windows motor
- Fuel level
- Lights
- Interior and exterior temperature
- Wheel pressure
- Speed
- Music
- Free hands

Why is this type of communication introduced in vehicles?

The main advantage for which the CAN Network was installed is that the number of cables and connections inside the vehicle is reduced around "45%". It is a type of serial and digital signal (i.e. they travel as 0 and 1).

In order to achieve this, each unit (or computer) has a decoder that "converts" the signals into 0 and 1. These signals have two wires, CAN H (CAN "High" and CAN L ("Low", which operates between 1.5 to 2.5 V).

This used to isolate each other to prevent electrical noise or electromagnetic waves from interfering with the signal.

How fast is CAN information transferred?

The speed with which the data is transmitted is: CAN_H (or high) = 500 Kbits/s and CAN_L (or low) 125 Kbits/s and at the end of each cable line (inside each computer) a resistance between them of 120 ohms.
