# Supplementary material for: EEG-Based Tool for Prediction of University Students’ Cognitive Performance in the Classroom
Source: Brain Sci. 2021 May 26;11(6):698. doi: 10.3390/brainsci11060698 (PMC8227309; doi:10.3390/brainsci11060698)
Supplement: Supplementary file 1 [file brainsci-11-00698-s001.zip › Supplementary Material/LT Text Spanish.pdf]

RED CAN

- También conocida “CAN BUS”
- Todas las unidades de control, se comunican mediante esta red (Controller Area Network)

¿Qué es una Red CAN? (Controller Area Network)
Es un protocolo de comunicación entre módulos electrónicos**.** Esta comunicación permite transportar gran cantidad de datos por un solo cable.

¿Quién y cuándo se creó la Red?
Incorporada en un vehículo por primera vez en 1987 por Intel y Philips, en la actualidad lo poseen todos los vehículos.

¿Cuáles son los componentes que tiene la Red CAN?

- **Controlador (Transmisor y Receptor):**
  - Estos componentes son los módulos (o computadoras).
  - Este circuito recibe, codifica y envía la información.
  - Manejan un valor de corriente muy bajo, en general es 0.5 A o menos.
- **Cables multiplexados:**
  - Son los cables encargados de llevar esta información.
  - Filtran el ruido eléctrico.
- **Terminador o Cierre:**
  - Elementos resistivos que se utilizan para cerrar y adecuar el funcionamiento del sistema.

Este tipo de comunicación permite una conexión entre las diversas "computadoras" que tiene el vehículo de manera muy eficiente; a todo este conjunto se le llama sistema multiplexado de comunicación.
Deben seguir ciertas reglas o protocolos, a este protocolo se le llama CAN (Controller Area Network) Algunas informaciones que van por esta red son:

- Presión de aceite de motor
- Levanta cristales
- Nivel de combustible
- Luces
- Temperatura interior o exterior del vehículo
- Presión de neumáticos
- Velocidad crucero
- Música
- Manos libres

¿Por qué se introduce este tipo de comunicación en los vehículos?

La principal ventaja por la cual se colocó la Red CAN es que se llega a reducir alrededor de un "45%" la cantidad de cables y conexiones dentro del vehículo. Es un tipo de señal serial y digital (es decir, van 0 y 1).

Para poder lograr esto, cada unidad (o computadora) tiene un decodificador que "convierte" las señales en 0 y 1.
Estas señales poseen dos cables, CAN H (CAN alto o "High" y CAN L (Bajo o "Low", que opera entre 1.5 a 2.5 V).

Esto es para justamente aislarse mutuamente para evitar que el ruido eléctrico u ondas electromagnéticas interfieran en la señal.

¿A qué velocidad se transfiere la información Can?

La velocidad con la que se transmiten los datos es: CAN_H (o alto) = 500 Kbits/s y CAN_L (o bajo) 125 Kbits/s y al final de cada línea de cables (dentro de cada computador) una resistencia entre ellos de 120 ohms.
